# Homogeneous and Multiphase Analysis of Nanofluids Containing Nonspherical MWCNT and GNP Nanoparticles Considering the Influence of Interfacial Layering

**DOI:** 10.3390/nano11020277

**Published:** 2021-01-21

**Authors:** Tehmina Ambreen, Arslan Saleem, Cheol Woo Park

**Affiliations:** School of Mechanical Engineering, Kyungpook National University, 80 Daehakro, Bukgu, Daegu 41566, Korea; tehminaambreen91@gmail.com (T.A.); arslansaleem@knu.ac.kr (A.S.)

**Keywords:** nanofluids, interfacial nanolayering, nonspherical nanoparticles, homogeneous, Eulerian–Eulerian, Lagrangian–Eulerian

## Abstract

The practical implication of nanofluids is essentially dependent on their accurate modelling, particularly in comparison with the high cost of experimental investigations, yet the accuracy of different computational approaches to simulate nanofluids remains controversial to this day. Therefore, the present study is aimed at analysing the homogenous, multiphase Eulerian–Eulerian (volume of fluid, mixture, Eulerian) and Lagrangian–Eulerian approximation of nanofluids containing nonspherical nanoparticles. The heat transfer and pressure drop characteristics of the multiwalled carbon nanotubes (MWCNT)-based and multiwalled carbon nanotubes/graphene nanoplatelets (MWCNT/GNP)-based nanofluids are computed by incorporating the influence of several physical mechanisms, including interfacial nanolayering. The accuracy of tested computational approaches is evaluated by considering particle concentration and Reynolds number ranges of 0.075–0.25 wt% and 200–470, respectively. The results demonstrate that for all nanofluid combinations and operational conditions, the Lagrangian–Eulerian approximation provides the most accurate convective heat transfer coefficient values with a maximum deviation of 5.34% for 0.25 wt% of MWCNT–water nanofluid at the largest Reynolds number, while single-phase and Eulerian–Eulerian multiphase models accurately estimate the thermal fields of the diluted nanofluids at low Reynolds numbers, but overestimate the results for denser nanofluids at high Reynolds numbers.

## 1. Introduction

Nanofluids are nanotechnology-based novel fluids of ultrahigh thermal efficiency. Over the past several decades, these thermal fluids have gained overwhelming attention in relevance to their potential applications in microelectronics, transportation, refrigeration, solar, nuclear and space technologies [1,2]. Even at a low concentration, nanoparticles possess high effective surface area and therefore exhibit exceptional thermal efficiency, flexible thermophysical properties, suspension stability and enlarged solid–particle interface for maximum interphase heat exchange. Additionally, the reduced tendency of sedimentation, channel clogging and negligible pressure drop make nanofluids appealing for the advanced thermal engineering systems [3,4,5,6]. The hydrothermal performance of the nanofluids is characterised by nanoparticle loading, morphology, hosting fluid thermophysical properties and operational temperature [7,8,9,10]. The assessment of hydrothermal characteristics of nanofluids for practical applications is essentially dependent on their accurate modelling, particularly in comparison with high-cost experimental investigations. The accurate simulation of nanofluids requires an inclusive understanding of the underlying physical phenomena responsible for the enhanced thermal activity of these wonder fluids [11,12]. Nanoparticle Brownian motion, drag and lift forces, thermophoresis, thermal boundary layer disruption and liquid molecular layering around nanoparticles are several of the extensively accepted potential mechanisms [13,14,15,16]. 

Among the different computational techniques, the Navier–Stokes-based homogeneous and multiphase approaches are the most commonly adopted ones to simulate nanofluid flow and heat transfer characteristics. In the single-phase approach, nanofluids are presumed as a homogenous mixture of nanoparticles and hosting fluid with negligible slip in between. This homogenous solution is assumed to exhibit enriched thermophysical properties that are assessed using experimental observations or theoretical models. Unlike the single-phase model, the nanoparticles and the hosting fluids are modelled as distinct phases in the multiphase numerical approaches and their mutual interactive forces are computed. The multiphase approaches are classified as Eulerian–Eulerian and Lagrangian–Eulerian based on treatment of nanoparticles and liquid phases. In the Lagrangian–Eulerian approach, the nanoparticles are treated as discrete entities and the governing equations of the liquid phase are simulated in the continuum approximation. The Eulerian–Eulerian approximation is based on the assumption of continuous interpenetration of the nanoparticles and the liquid phases. The latter approach comprises three numerical models of the volume of fluid (VOF), mixture and the Eulerian models. In the VOF model, the two phases are approximated as immiscible regimes and their interphase is traced. The mixture model considers that individual phases of the solution comprise their slip velocities and volume concentration fields. However, a single set of governing equations is solved for all of the phases, whereas the transport equations of each phase are solved independently in the Eulerian model and the coupling between all phases is achieved with the pressure and interphase exchange coefficients. 

Concerning the accuracy of homogeneous and multiphase approaches to compute nanofluids, so far, the literature contains immense controversies and debate about the selection of an accurate model. The homogeneous or single-phase approximation of nanofluid, credited with simplicity and cost-effectiveness, is the most extensively utilised technique [17,18,19,20,21,22,23]. Several comparative reports also emphasised the accuracy of this approach [24,25,26,27,28]. However, a group of researchers condemned homogenous approximation of highly concentrated nanofluids due to the significant influence of interphase interactive forces that are neglected in this approach [2,29,30,31,32,33,34,35,36,37]. Analysing forced convection of Al_2_O_3_/H_2_O, TiO_2_/H_2_O and Cu/H_2_O nanofluids, Nishat et al. [38] demonstrated that the homogeneous model accurately predicts the highly concentrated nanofluids (0.5% ≤ *φ* ≤ 2%) compared with the Lagrangian–Eulerian model. Similar contradictory statements are witnessed related to multiphase numerical modelling of the nanofluids. Majid et al. [39] evaluated the Eulerian and mixture models to assess the hydrothermal characteristics of the Al_2_O_3_/H_2_O (0.1% ≤ *φ* ≤ 2%) nanofluid and demonstrated that the analogous results estimated by both of the models were consistent with the experimental data. However, in an assessment of homogeneous, multiphase mixture and Eulerian models for simulating hydrothermal aspects of the Al_2_O_3_/H_2_O (0.6% ≤ *φ* ≤ 1.6%) nanofluid, Göktepe et al. [40] criticised the mixture model for underperforming compared with the Eulerian model in terms of accuracy. On the contrary, Lotfi et al. [41], Ehsan et al. [37] and Behroyan et al. [28] critiqued the capability of the Eulerian model to simulate the thermal behaviours of the nanofluids. Lotfi et al. [41] and Behroyan et al. [28] stated an underestimation of the heat transfer enhancement of Al_2_O_3_/H_2_O (2% ≤ *φ* ≤ 7%) and Cu/H_2_O (1% ≤ *φ* ≤ 2%) nanofluids, respectively, whereas Ehsan et al. [37] overestimated the convective heat transfer coefficient results of TiO_2_/H_2_O (1% ≤ *φ* ≤ 2.3%) nanofluid when computed utilising the Eulerian approach. In a comparative evaluation of single-phase and Eulerian–Eulerian models, Akbari et al. [25,31] stated that Eulerian, mixture and VOF models overpredict the convective heat transfer coefficient of highly concentrated Al_2_O_3_/H_2_O (0.6% ≤ *φ* ≤ 1.6%) nanofluid. For the Lagrangian–Eulerian approach, several reports endorsed its superiority [32,33,34,36,42], but a few conflicting reports claimed otherwise [26]. The possible justifications of these contradictions are the insufficient nanofluid-related numerical investigations, lack of understanding of physical phenomena responsible for the enhanced thermal activity of nanofluids and inaccurate modelling. Another major influencing factor is the anomalies in the experimental data used for the validation of different numerical models. Considering the fact that thermophysical properties of nanofluids are immensely affected by the nanofluids’ stability, nanoparticle agglomeration or sedimentation in unstable nanofluid samples may mislead the evaluated results. 

In view of the above controversies, a recent review emphasised conducting systematic investigations on the single- and multiphase modelling of nanofluids by incorporating all of the correlated physical mechanisms to establish fundamental results [43,44]. The report also accentuated evaluating the accuracy of different computational models to predict the hydrothermal characteristics of the nonspherical nanoparticles, given that all of the previous relevant studies are based on spherical approximation of nanoparticles. Therefore, addressing this research gap, in the present study, we investigate the accuracy of homogenous, Eulerian–Eulerian (VOF, mixture, Eulerian) and Lagrangian–Eulerian approaches for simulating the hydrothermal characteristics of the MWCNT–H_2_O and MWCNT/GNP–H_2_O hybrid nanofluids by incorporating several physical mechanisms, including interfacial nanolayering. The influence of nanolayering around nanoparticles is examined by introducing an effective volume fraction of nanoparticles that defines the combined effects of nanoparticle volume fraction and surfactants on the rheological properties of the solution.

## 2. Problem Description

**Figure 1:**
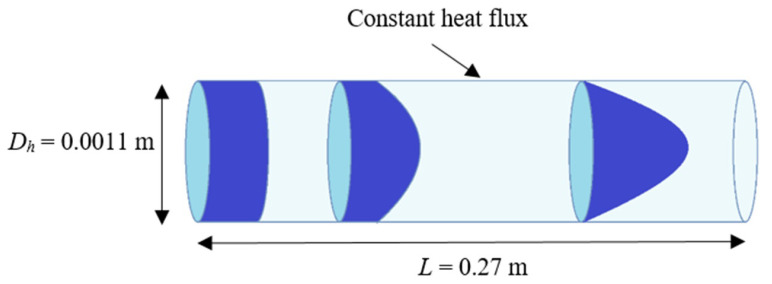
Physical description of problem.

The forced convection of aqueous-based nanofluids containing suspensions of nonspherical MWCNT and MWCNT/GNP hybrid nanoparticles is simulated in a horizontal minichannel subjected to a constant heat flux of 11,800 W/m^2^ (Figure 1). The probed range of the Reynolds number is 200 ≤ *Re* ≤ 470 and the flow is presumed as developing and fully developed. The considered nanoparticle weight concentrations are 0.075% ≤ *ψ* ≤ 0.25% for the MWCNT-based nanofluids, whereas each sample contained an additional *ψ* = 0.035% of GNP nanoparticles for the case of MWCNT/GNP hybrid nanofluids. The MWCNT and GNP nanoparticles are assumed to be dispersed homogenously into the aqueous solution without any surfactant. The MWCNT nanoparticles are approximated as tubular-shaped with an average length and diameter of 3 um and 15 nm, respectively. The averaged diameter and thickness of disc-shaped GNP nanoparticles are 7 nm and 15 um, respectively. The minichannel hydraulic diameter and length are *D_h_* = 0.0011 m and *L* = 0.27 m, respectively. The accuracy of the computed results is assessed in comparison with the experimental conditions specified by Hussein et al. [45] due to the availability of rheological properties of the nanofluid. All the specifics of the physical model and nanofluid combinations are kept in accordance with the experimental study of Hussein et al. [45] to perform a comprehensive comparison of the tested numerical models.

## 3. Mathematical Formulation

### 3.1. Single-Phase Model

The single-phase modelling of nanofluids is based on the assumption that the solution is a single homogenous fluid of enhanced thermophysical properties. The nanoparticle and dispersion phases are assumed to be thermally and hydrodynamically at equilibrium with each other and, consequently, exhibit negligible interphase momentum and energy exchange. The accuracy of this approach is predominantly dependent on the accurate prediction of the thermophysical properties of the nanofluids, which are therefore presently estimated using the same property correlations utilised in the respective experimental study [45]. The single-phase model’s governing equations are

(1) $$\nabla \cdot \left(\rho_{nf}\vec{V}\right)=0$$

(2) $$\nabla \left(\rho_{nf}\vec{V}\vec{V}\right)=-\nabla P+\nabla \cdot \left(\mu_{nf}\nabla \vec{V}\right)+\rho_{nf}g$$

(3) $$\nabla \cdot \left(\rho_{nf}\vec{V}H\right)=\nabla \cdot \left(k_{nf}\nabla T\right)$$

The density ($\rho_{nf}$) and specific heat (${\left(c_{p}\right)}_{nf}$) of the nanofluids are estimated by Equations (4) [14] and (5) [46], respectively, while Equations (6) [47] and (7) [48] define the temperature-dependent viscosity ($\mu_{nf}$) and thermal conductivity ($k_{nf}$) of the nanofluids, respectively.

(4) $$\rho_{nf}=\varphi \rho_{s}+\left(1-\varphi \right)\rho_{f}$$

(5) $${\left(c_{p}\rho \right)}_{nf}=\varphi {\left(c_{p}\rho \right)}_{s}+\left(1-\varphi \right){\left(c_{p}\rho \right)}_{f}$$

(6) $$\mu_{nf}=\left(1+2.5\varphi +6.2\varphi^{2}\right)\mu_{f}\left(T\right)$$

(7) $$k_{nf}=\left(\frac{k_{s}+(n-1)k_{f}\left(T\right)+\left(n-1\right)\left(k_{s}-k_{f}\left(T\right)\right)\varphi }{k_{s}+(n-1)k_{f}\left(T\right)-\left(k_{s}-k_{f}\left(T\right)\right)\varphi }\right)k_{f}\left(T\right)$$

Here, *n* = 3/*ϖ* is the shape factor of the nanoparticle and *ϖ* denotes the sphericity of the particles. Sphericity is the ratio of the surface area of a sphere with the volume equivalent to the average particle to the surface area of the particle. The value of *s* is 1 and 0.5 for the cases of spherical and cylindrical nanoparticles.

The volume (*φ*) and mass fractions (*ψ*) of the nanoparticles in the solution are associated as

(8) $$\varphi =\frac{\psi \rho_{f}}{\rho_{s}+\psi \rho_{f}-\psi \rho_{s}}$$

The effective volume fraction *φ* is defined [49] as

(9) $$\varphi =\frac{\eta_{r}-1}{2.5}$$

The relative viscosity *η_r_* is the ratio of nanofluid viscosity (*μ_nf_*) to the viscosity of the base fluid ($k_{f}$).

### 3.2. Eulerian–Eulerian Model

The investigated Eulerian–Eulerian models include mixture, VOF and Eulerian models based on the coupling between the phases and considered interphase interactions. 

#### 3.2.1. Mixture Model

In the mixture model, the continuity, momentum and energy equations are solved for the mixture by assuming a weak interphase coupling. Each phase is presumed to exhibit its peculiar velocity field vector and volume fraction. The thermophysical properties of the mixture are defined as the weighted average of all the phases corresponding to their volume fraction. The primary phase (liquid) influences the secondary phases (nanoparticles) via drag force while the secondary phases affect the primary phase by reducing its mean momentum. The governing equations incorporated in this model are

(10) $$\nabla \cdot \left(\rho_{nf}{\vec{V}}_{nf}\right)=0$$

(11) $$\nabla \left(\rho_{nf}{\vec{V}}_{nf}{\vec{V}}_{nf}\right)=-\nabla P+\nabla \cdot \left(\mu_{nf}\nabla {\vec{V}}_{nf})+\nabla \cdot (\varphi_{f}\rho_{f}{\vec{V}}_{dr,f}{\vec{V}}_{dr,f}+\varphi_{s}\rho_{s}{\vec{V}}_{dr,s}{\vec{V}}_{dr,s}\right)+\rho_{nf}g$$

(12) $$\nabla \cdot \left(\varphi_{f}{\vec{V}}_{f}\rho_{f}C_{p_{f}}T_{f}+\varphi_{s}{\vec{V}}_{s}\rho_{s}C_{p_{s}}T_{s}\right)=\nabla \cdot \left(k_{nf}\nabla T_{nf}\right)$$

The summation of volume fraction of all the phases is given by $\overset{n}{\underset{i=1}{\sum }}\varphi_{i}=1$, where *n* is the number of phases.

The nanofluid mixture velocity is determined using ${\vec{V}}_{m}=\frac{\varphi_{f}{\vec{V}}_{f}\rho_{f}+\varphi_{s}{\vec{V}}_{s}\rho_{s}}{\rho_{nf}}$ and the drift velocity of the *i*th phase is approximated as ${\vec{V}}_{dr,i}={\vec{V}}_{i}-{\vec{V}}_{nf}$. Any of the mixture properties (*J*) such as thermal conductivity, density and viscosity are determined as

(13) $$N=\overset{n}{\underset{i=1}{\sum }}\varphi_{i}J_{i}$$

(14) $$\nabla \cdot \left(\varphi_{s}\rho_{s}{\vec{V}}_{nf}\right)=-\nabla \cdot \left(\varphi_{s}\rho_{s}{\vec{V}}_{dr,s}\right)$$

The correlation (Equation (15)) of Manninen et al. [50] is used to estimate the slip velocity, that is, the relative velocity of nanoparticles w.r.t. base fluid (${\vec{V}}_{sf}={\vec{V}}_{s}-{\vec{V}}_{f}$):

(15) $${\vec{V}}_{fs}=\frac{\rho_{s}d_{s}^{2}}{18\mu_{f}F_{drag}}\frac{\rho_{f}-\rho_{nf}}{\rho_{f}}\vec{a}$$

where

(16) $$\vec{a}=\vec{g}-\left({\vec{V}}_{nf}\cdot \nabla \right){\vec{V}}_{nf}$$

Drag force is estimated by the correlation of Schiller and Naumann [51] as a function of the Reynolds number:

(17) $$F_{drag}=\{\begin{array}{c} 1+0.15Re_{s}^{0.687}\\ 0.0183Re_{s} \end{array}\;\;\;\;\;\;\;\;\;\;\begin{array}{c} Re_{s}\leq 1000\\ Re_{s}>1000 \end{array}$$

where

(18) $$Re_{s}=\frac{{\vec{V}}_{nf}d_{s}}{v_{nf}}$$

In Equation (18), $d_{s}$ denotes the nanoparticle diameter that is assumed as the diameter of a spherical particle with the equivalent surface area.

#### 3.2.2. Volume of Fluid Model

In this model, the interface between phases is tracked by solving a continuity equation for the volume fraction of the fluid phase. A momentum equation is solved to compute the velocity components shared by all phases. Similarly, a shared temperature is estimated from the single energy equation. The governing equations of the model are

(19) $$\nabla \cdot \left(\varphi_{f}\rho_{f}{\vec{V}}_{f}\right)=0$$

(20) $$\nabla \left(\rho_{nf}{\vec{V}}_{nf}{\vec{V}}_{nf}\right)=-\nabla P+\nabla \cdot \left(\mu_{nf}\nabla {\vec{V}}_{nf}\right)+\rho_{nf}g$$

(21) $$\nabla \cdot \left({\vec{V}}_{nf}\rho_{nf}C_{p_{nf}}T_{nf}\right)=\nabla \cdot \left(k_{nf}\nabla T_{nf}\right)$$

The summation of the volume fractions of all the phases is given by $\overset{n}{\underset{i=1}{\sum }}\varphi_{i}=1$, where *n* is the number of phases. Similar to the mixture model, the thermophysical properties of the solution are calculated by taking a weighted average of different phases based on their volume fractions (Equation (13)).

#### 3.2.3. Eulerian Model

In the Eulerian model, the coupling between the phases is presumed to be strong and pressure is shared by all the phases. Separate continuity, momentum and energy equations are simulated for all the primary and secondary phases. The interphase momentum transfer is given by the drag and Saffman lift force, whereas particle–particle interactions are defined in terms of collision forces. The volume of individual phases is calculated by integrating their volume fractions throughout the domain. The summation of the volume fractions of all the phases is given by $\overset{n}{\underset{i=1}{\sum }}\varphi_{i}=1$. The governing equations of the primary (liquid) and secondary (nanoparticle) phases are

(22) $$\nabla \cdot \left(\varphi_{f}\rho_{f}{\vec{V}}_{f}\right)=0$$

(23) $$\nabla \cdot \left(\varphi_{s}\rho_{s}{\vec{V}}_{s}\right)=0$$

(24) $$\nabla \left(\varphi_{f}\rho_{f}{\vec{V}}_{f}{\vec{V}}_{f}\right)=-\varphi_{f}\nabla P+\varphi_{f}\nabla \cdot \left(\mu_{f}\nabla {\vec{V}}_{f}\right)+\varphi_{f}\rho_{f}\vec{g}+F_{drag,f}+{\vec{F}}_{lift,f}$$

(25) $$\nabla \left(\varphi_{s}\rho_{s}{\vec{V}}_{s}{\vec{V}}_{s}\right)=-\varphi_{s}\nabla P+\varphi_{s}\nabla \cdot \left(\mu_{s}\nabla {\vec{V}}_{s}\right)+\varphi_{s}\rho_{s}\vec{g}+F_{drag,s}+{\vec{F}}_{lift,s}++{\vec{F}}_{col,s}$$

(26) $$\nabla \cdot \left(\varphi_{f}{\vec{V}}_{f}\rho_{f}C_{p_{f}}T_{f}\right)=\nabla \cdot \left(\varphi_{f}k_{f}\nabla T_{f}\right)-h_{v}\left(T_{s}-T_{f}\right)$$

(27) $$\nabla \cdot \left(\varphi_{s}{\vec{V}}_{s}\rho_{s}C_{p_{s}}T_{s}\right)=\nabla \cdot \left(\varphi_{s}k_{s}\nabla T_{s}\right)-h_{v}\left(T_{s}-T_{f}\right)$$

The lift ($F_{lift}$), collision ($F_{col,s}$) and the drag forces ($F_{drag}$) are estimated by Equations (28)–(31), respectively.

(28) $$F_{lift,f}=-0.5\rho_{s}\varphi_{f}\left({\vec{V}}_{s}-{\vec{V}}_{f}\right)\times \left(\nabla \times {\vec{V}}_{s}\right)$$

(29) $$F_{lift,s}=-0.5\rho_{f}\varphi_{s}\left({\vec{V}}_{f}-{\vec{V}}_{s}\right)\times \left(\nabla \times {\vec{V}}_{f}\right)$$

(30) $$F_{col,s}=\exp\left[-600\left(\varphi_{s}-0.376\right)\right]\left(\nabla \varphi_{s}\right)$$

(31) $$F_{drag,f}\;\;=F_{drag,s}=\frac{\varphi_{f}\varphi_{s}f_{D}18{µ}_{f}}{d_{s}^{2}}\left({\vec{V}}_{f}-{\vec{V}}_{s}\right)$$

(32) $$f_{D}=\frac{C_{D}Re_{s}}{24}$$

(33) $$C_{D}=\{\begin{array}{c} \frac{24\left(1+0.15Re_{s}^{0.687}\right)}{Re_{s}}\\ 0.44 \end{array}\;\;\;\;\;\;\;\;\;\;\begin{array}{c} Re_{s}\leq 1000\\ Re_{s}\;>1000 \end{array}$$

$F_{lift}$ is determined using the Drew and Lahey [52] equation (Equations (28) and (29)), whereas $F_{col,s}$ is estimated by the Bouillard et al. [53] correlation. In Equation (32), the drag coefficient ($f_{D}$) is estimated by the Schiller and Naumann [51] correlation. The Reynolds number of the secondary phase in Equation (33) is defined as

(34) $$Re_{s}\;=\frac{d_{s}\left|{\vec{V}}_{s}-{\vec{V}}_{f}\right|d_{k}\rho_{f}}{\mu_{f}}$$

The volumetric interphase heat exchange (Equations (26) and (27)) is calculated using the following equation:

(35) $$h_{v}=6k_{s}\varphi_{s}\varphi_{f}Nu_{s}/d_{s}^{2}\;$$

where $Nu_{s}$ is estimated using the Ranz and Marshall correlation [54]:

(36) $$Nu_{s}=2+0.6Re_{s}^{0.5}Pr_{s}^{0.33}$$

In all the Eulerian–Eulerian models, the diameter of the spherical particles with the equivalent surface area is defined. 

### 3.3. Lagrangian–Eulerian Model

In the Lagrangian–Eulerian model, the trajectories of nanoparticles are traced in the Lagrangian approach while the governing equations of the primary phase are computed in the continuum (Eulerian) approximation. The momentum and energy exchange between the primary and particulate phases are simulated by incorporating source terms in the momentum ($S_{m}$) and energy ($S_{e}$) equations of the primary phase. The momentum interactions include particle Brownian motion, drag and Saffman lift forces, thermophoresis, virtual mass, gravity and pressure-gradient-induced forces. The interparticle collisions are supposed to be negligible considering the small weight concentration of nanoparticles. The MWCNT and GNP nanoparticles are assumed as tubular- and thin-disc-shaped, respectively. The governing equations of the primary phase are

(37) $$\nabla \cdot \left(\rho_{f}{\vec{V}}_{f}\right)=0$$

(38) $$\nabla \left(\rho_{f}{\vec{V}}_{f}{\vec{V}}_{f}\right)=-\nabla P+\nabla \cdot \left(\mu_{f}\nabla {\vec{V}}_{f}\right)+S_{m}$$

(39) $$\nabla \cdot \left({\vec{V}}_{f}\rho_{f}C_{p_{f}}T_{f}\right)=\nabla \cdot \left(k_{f}\nabla T_{f}\right)+S_{e}$$

where

(40) $$S_{m}=\sum^{\;}n_{s}\frac{m_{s}}{\delta V}\;\frac{dV_{s}}{dt}$$

(41) $$\frac{dV_{s}}{dt}=F_{drag}+F_{gravity}+F_{Brownian}+F_{thermophoresis}+F_{lift}+F_{virtual}+F_{pressure}$$

where $F_{drag}$ and $F_{lift}$ are estimated by the Stokes drag and Saffman’s lift [55] correlations, respectively.

(42) $$F_{drag}=\frac{18\mu_{f}}{d_{p}^{2}\rho_{p}C_{c}}\left(V_{f}-V_{s}\right)$$

(43) $$F_{gravity}=\frac{g\left(\rho_{s}-\rho_{f}\right)}{\rho_{s}}$$

(44) $$F_{Brownian}=\;\zeta_{i}\sqrt{\frac{\pi }{\Delta t}\frac{216\nu_{f}K_{B}T}{\pi^{2}\rho_{f}d_{p}^{5}{\left(\frac{\rho_{s}}{\rho_{f}}\right)}^{2}C_{c}}}$$

(45) $$F_{thermophoresis}=\frac{7.02\pi d_{s}\mu_{f}^{2}\left(K+2.18K_{n}\right)}{m_{s}\rho_{f}T\left(1+3.42K_{n}\right)\left(1+2K+4.36K_{n}\right)}\times \frac{\partial T}{\partial x}$$

(46) $$F_{lift}=\frac{5.188v_{f}^{0.5}\rho_{f}d_{ij}}{\rho_{s}d_{s}{\left(d_{lk}d_{kl}\right)}^{0.25}}\left(V_{f}-V_{s}\right)$$

(47) $$F_{virtual}=0.5\frac{\rho_{f}}{\rho_{s}}\frac{d}{dt}\left(V_{f}-V_{s}\right)$$

(48) $$F_{pressure}=\left(\frac{\rho_{f}}{\rho_{s}}\right)V_{s}\frac{dV_{f}}{dx}$$

In Equation (45), $K=\frac{k}{k_{s}}.$ The $d_{ij}$ and $K_{n}=\frac{2\lambda }{d_{s}}$ are the deformation tensor and Knudsen number, respectively. $\zeta_{i}$ in Equation (44) is the zero-mean, unit-variance-independent Gaussian random numbers. The $C_{c}$ is the Cunningham correction factor of the spherical nanoparticles. The modified Cunningham correction factors for cylindrical (MWCNT) and thin-disc (GNP)-shaped nanoparticles are given in Equations (49) and (50) [56], respectively.

(49) $$C_{c}=\frac{3d_{L}\left[ln\left(2\beta \right)-0.72\right]}{2}\;$$

(50) $$C_{c}=\frac{3\pi }{8}$$

In the above equations, *β* denotes the aspect ratio of the particle length to diameter. The energy source term $S_{e}$ in Equation (39) is defined as

(51) $$S_{e}=\sum^{\;}n_{s}\frac{m_{s}}{\delta V}\;C_{p}\frac{dT_{s}}{dt}$$

(52) $$m_{s}C_{s}\frac{dT_{s}}{dt}=h_{i}A_{s}\left(T_{f}-T_{s}\right)$$

(53) $$\;h_{i}=\frac{Nu_{s}k_{f}}{d_{s}}$$

where $Nu_{s}$ is estimated by incorporating the Ranz and Marshall model (Equation (36)) [54].

For all the tested models, the thermophysical properties of the nanoparticles are listed in Table 1. Base fluid (primary phase for multiphase models) temperature-dependent properties are assessed using the following expressions:

(54) $$\rho_{f}=\;2446-20.674T+0.11576\;T^{2}-3.12895*10^{-4}T^{3}-2.0546*10^{-10}T^{5}$$

(55) $${\left(C_{p}\right)}_{f}=\exp\left(\frac{8.29041-0.012557T}{1-\left(1.52373*10^{-3}\right)T}\right)$$

(56) $${µ}_{f}=2.414*10^{-5}*10^{\left(\frac{247.8}{T-140}\right)}$$

(57) $$k_{f}=\;0.5706+1.756*10^{-3}\;T\;-6.46*10^{-6}\;T^{2}$$

In the single-phase model, the nanoparticle shape effect is modelled through the shape factor (*n*) in the effective thermal conductivity of the nanofluids (Equation (7)). The nanoparticles are assumed as spherical-shaped with the surface area equivalent to the considered nanoparticles in all the Eulerian–Eulerian models (mixture, VOF and Eulerian), whereas in the Lagrangian–Eulerian model, the nanoparticle shape is modelled through the modified Cunningham correction factor (Equations (49) and (50)) in the particle drag (Equation (42)) and Brownian motion (Equation (44)).

## 4. Numerical Details and Grid Independency

The numerical analysis is conducted by employing the finite-volume-method-based commercial software ANSYS Fluent [58]. The pressure-based coupled algorithm is used for pressure–velocity coupling and the second-order upwind scheme is utilised for the discretisation of the convective terms [58]. To track the secondary-phase volume fraction, the implicit formulation is used in VOF and Eulerian models. The first-order upwind scheme is applied for the discretisation of volume fraction in both mixture and Eulerian models while, in the case of the VOF model, a compressive scheme is incorporated. The nanoparticles are introduced into the computational flow domain as surface injection type in the Lagrangian–Eulerian model [10,42,59]. The trapezoidal high-order scheme and implicit low-order scheme are implemented for nanoparticle tracking. 

**Figure 2:**
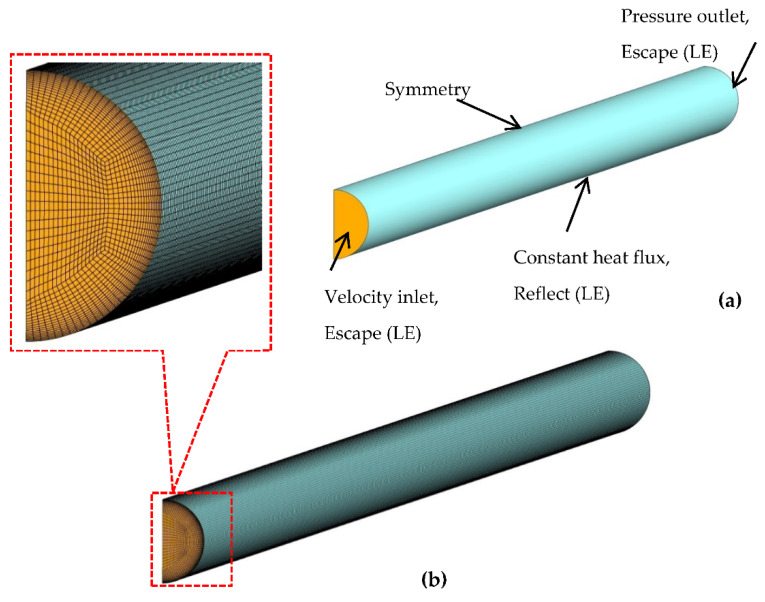
(**a**) Boundary conditions; (**b**) mesh topology of the computational domain.

At the inlet, outlet and wall of the minichannel, boundary conditions of velocity inlet, pressure outlet and constant heat flux are respectively defined in accordance with their respective experimental test conditions [45]. Additional escape boundary conditions at the channel inlet and outlet and reflect boundary conditions at the channel wall are defined for the nanoparticle tracing in the Lagrangian–Eulerian model. Considering the symmetry of the computational domain, half of the channel is modelled by implying symmetry boundary condition (see Figure 2). Residuals of volume fraction, continuity, momentum and energy equations are ensured to be less than $10^{-7}$ for the convergence criteria. 

**Figure 3:**
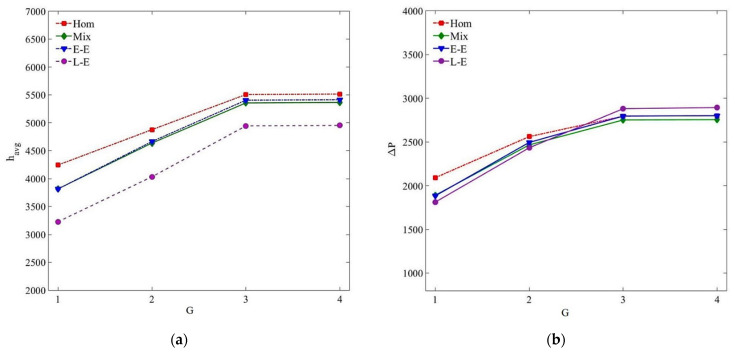
Grid sensitivity analysis for the forced convection of MWCNT/GNP–H_2_O hybrid nanofluid (*ψ* = 0.25%, *Re* = 470): (**a**) average heat transfer coefficient; (**b**) pressure drop.

A structured hexahedral grid is generated over the whole computational domain using the ICEM CFD package as depicted in Figure 2. The mesh is refined near the channel wall to capture the thermal–viscous boundary layer. Four grid sizes (G1–G4) of different resolutions are tested for the forced convection of the MWCNT/GNP–H_2_O hybrid nanofluid (*ψ* = 0.25%, *Re* = 470) by utilising all the tested single and multiphase models to ensure the grid-insensitive solution. The specifics of the tested mesh sizes are listed in Table 2. The results approximated by different grid sizes are compared in terms of average heat transfer coefficient (*h_avg_*) and pressure drop (Δ*P*) as illustrated in Figure 3. The results verify that *h_avg_* and Δ*P* values show nearly constant values when computed utilising grid G3 and G4 with the maximum percentage difference less than 0.5%. Thereby, grid G3 is utilised for all the subsequent computations (Y+ < 1) to assure solution accuracy at the expense of minimal computational cost. 

## 5. Results and Discussion

In this section, the accuracies of the single-phase and multiphase Eulerian–Eulerian (VOF, mixture, Eulerian) and Lagrangian–Eulerian approaches for simulating the nonspherical MWCNT and MWCNT-/GNP-nanoparticles-based nanofluids are evaluated. In all the tested models, the influence of nanolayering around the nanoparticles is incorporated in terms of effective volume fraction that describes the combined effects of nanoparticle volume fraction and surfactants on the rheological properties of the solution. The range of Reynolds number and nanoparticle weight concentrations are 200 ≤ *Re* ≤ 470 and 0.075% ≤ *ψ* ≤ 0.25%, respectively. The accuracy of the computed hydrothermal (average and local heat transfer coefficient, pressure drop) results is assessed through comparison with the relevant experimental results [45].

### 5.1. Validation of the Homogenous Model

The numerically calculated local Nusselt number results (along the channel wall) of the water (primary phase) are validated with the theoretical correlation of the Shah and London [60] (Equation (58)) for laminar flow under the boundary condition of constant heat flux at *Re* = 470:

(58) $$\left\{ \begin{array}{ccc} Nu_{x}=3.302x_{+}^{-1/3} & for & x_{+}\leq 0.00005\\ Nu_{x}=1.302x_{+}^{-1/3}-0.5 & for & 0.00005\leq x_{+}\leq 0.0015\\ Nu_{x}=4.362+8.68{\left(10^{3}x_{+}\right)}^{-0.506}e^{-41x_{+}} & for & x_{+}\geq 0.0015 \end{array}\right.$$

where

(59) $$x_{+}=\frac{X}{D_{h}Re\;Pr}$$

**Figure 4:**
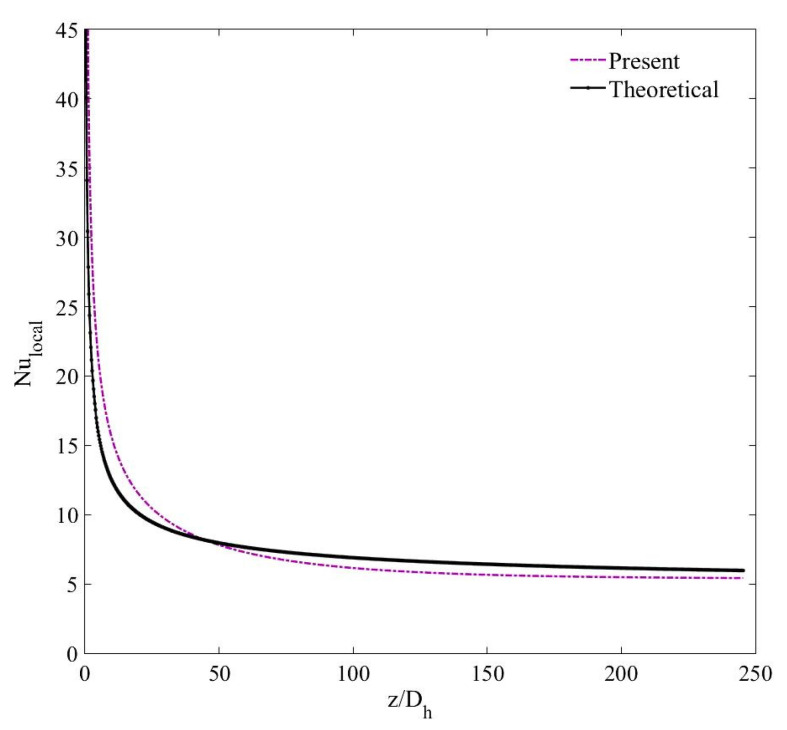
Validation of the computed local Nusselt number with the Shah and London correlation [60].

**Figure 5:**
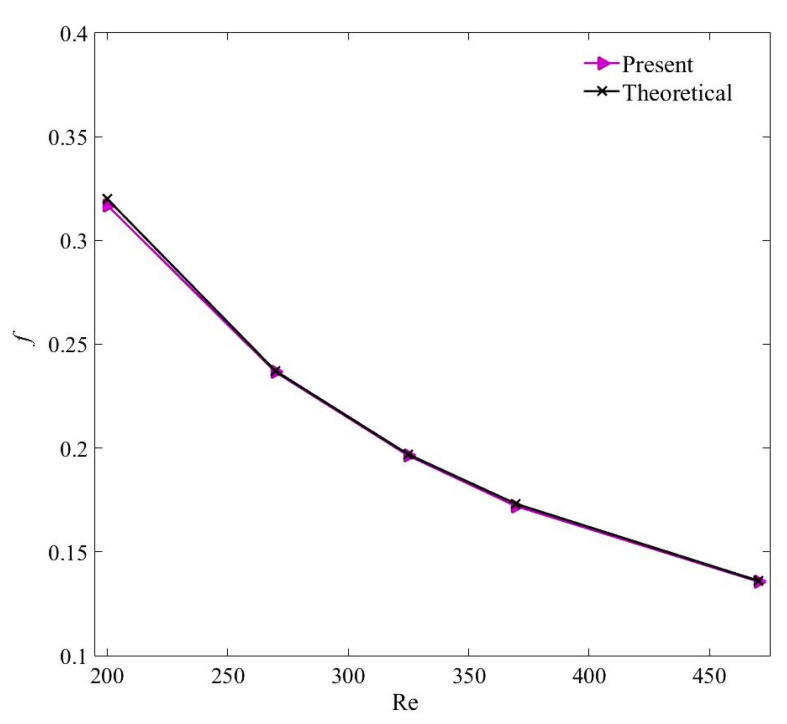
Validation of the computed friction factor with the Darcy friction factor equation [61].

In addition, the results of friction factor in fully developed flow region of the channel are compared with those estimated with the Darcy friction factor (f=64/Re) correlation [61]. Figure 4 and Figure 5 validate that the numerical results are in acceptable agreement with the theoretical values with a maximum percentage deviation of 10.6% and 1.9% for the local Nusselt number and friction factor, respectively. The average percentage difference between the computed and theoretical values of the local Nusselt number is 3.1%. 

### 5.2. Heat Transfer Characteristics 

**Figure 6:**
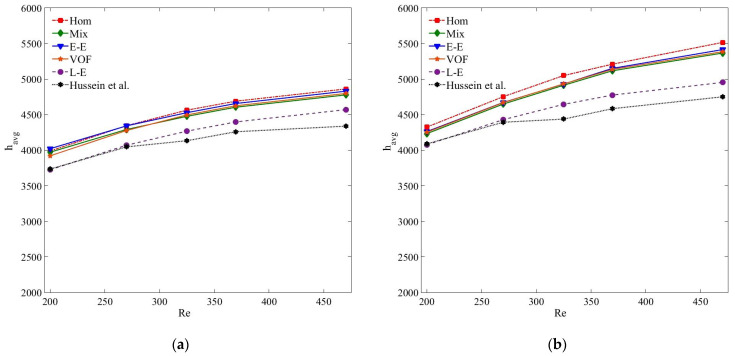
(**a**) Average heat transfer coefficient (*h_avg_*) of MWCNT–H_2_O (*ψ* = 0.25%) nanofluid; (**b**) average heat transfer coefficient (*h_avg_*) of MWCNT/GNP–H_2_O (*ψ* = 0.25%) nanofluid.

Figure 6 presents the average convective heat transfer coefficient (*h_avg_*) results of MWCNT–H_2_O and MWCNT/GNP–H_2_O nanofluids predicted using single and multiphase models for the nanoparticle concentrations of 0.25%. The figures illustrate that at *ψ* = 0.25%, the multiphase Eulerian–Eulerian (mixture, VOF and Eulerian) and single-phase models overestimate the *h_avg_* results of both nanofluid samples. Such overprediction of the thermal results computed by the Eulerian–Eulerian model is also highlighted in literature and was correlated with the overestimation of the volume-weighted average thermal conductivity of nanofluids [42,62]. All of the Eulerian–Eulerian models estimate quite similar values, whereas those of the single-phase model are slightly larger. The deviation of the Eulerian–Eulerian and single-phase models is more pronounced at high Reynolds numbers when the interphase interactions intensify, i.e., the percentage difference of *h_avg_* of MWCNT–H_2_O (*ψ* = 0.25%) nanofluid with the experimental data is 6.8% at *Re* = 200, which increases to 12.1% at *Re* = 470. Similarly, the percentage difference between the experimental and numerical values of Eulerian–Eulerian and single-phase models is more significant for the MWCNT/GNP–H_2_O hybrid nanofluid than for the MWCNT/H_2_O. This trend can be explained as the outcome of the stronger interphase interactions provided by the larger nanoparticle concentrations, given that the hybrid nanofluid comprises an additional 0.035 wt% of the GNP nanoparticles. Of all the single and multiphase approaches, the Lagrangian–Eulerian treatment of nanofluids results in the most accurate values with the maximum deviations of 5.34% and 4.9% for the MWCNT and MWCNT/GNP nanofluids, respectively, at *Re* = 470.

**Figure 7:**
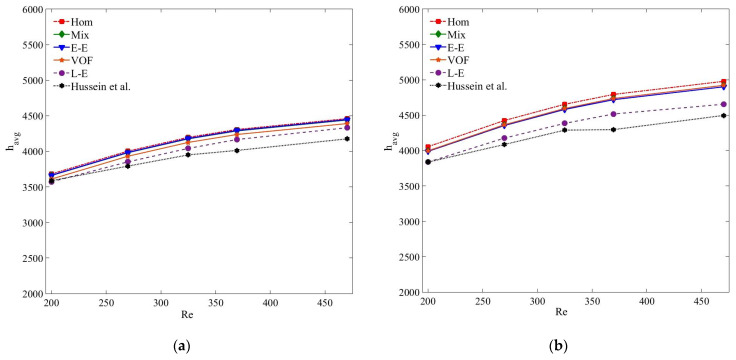
(**a**) Average heat transfer coefficient (*h_avg_*) of MWCNT–H_2_O (*ψ* = 0.125%) nanofluid; (**b**) average heat transfer coefficient (*h_avg_*) of MWCNT/GNP–H_2_O (*ψ* = 0.125%) nanofluid.

**Figure 8:**
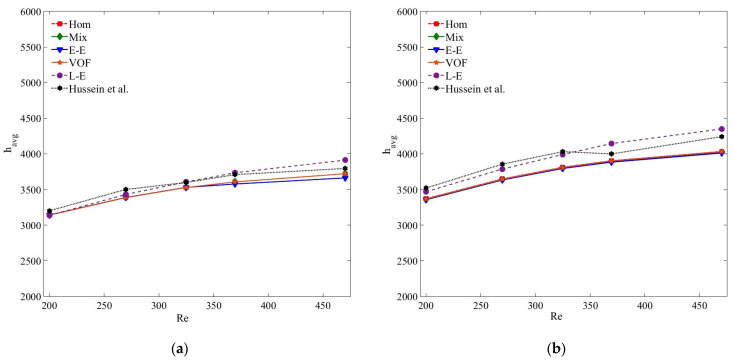
(**a**) Average heat transfer coefficient (*h_avg_*) of MWCNT-H_2_O (*ψ* = 0.075%) nanofluid; (**b**) average heat transfer coefficient (*h_avg_*) of MWCNT/GNP–H_2_O (*ψ* = 0.075%) nanofluid.

**Figure 9:**
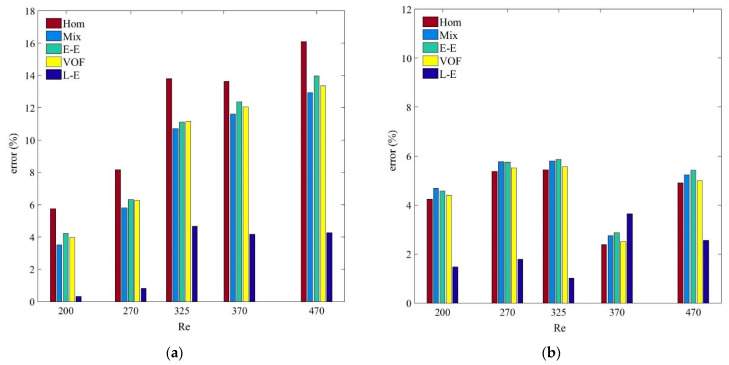
(**a**) Deviation of single-phase and multiphase models for (*h_avg_*) of MWCNT/GNP-H_2_O (*ψ* = 0.25%) nanofluid; (**b**) deviation of single-phase and multiphase models for (*h_avg_*) of MWCNT/GNP–H_2_O (ψ = 0.075%) nanofluid.

Figure 7 and Figure 8 reflect the *h_avg_* results of both the nanoparticle samples for the nanoparticle concentrations of 0.125% and 0.075%, respectively. It is apparent from the figures that all the Eulerian–Eulerian models compute identical yet slightly overestimated results. The accuracy of the tested models for predicting *h_avg_* values is in the order of Lagrangian–Eulerian, Eulerian–Eulerian and single-phase models. However, comparing the *h_avg_* values at *ψ* = 0.25%, the deviation between the experimental and numerical results decreases for small nanoparticle concentrations as an outcome of insignificant interphase interactions (Figure 9). At *Re* = 470, the percentage differences between the experimental and numerical results computed by the single, mixture, VOF, Eulerian and Lagrangian–Eulerian models are 6.7%, 6.3%, 6.4%, 5.5% and 3.7%, respectively, for the MWCNT-based nanofluids at *ψ* = 0.125%. This deviation reduces to 1.9%, 2%, 3.4%, 1.9% and 3.1%, respectively, for particle concentration *ψ* = 0.075%. Similarly, for MWCNT/GNP-based (*ψ* = 0.125%, *Re* = 470) nanofluid, the percentage errors between the experimental and computed results are 10.73%, 9.5%, 9%, 9.4% and 3.5% for the single, mixture, VOF, Eulerian and Lagrangian–Eulerian models, respectively. This deviation becomes insignificant with 4.9%, 5.23%, 5.43%, 5% and 2.56%, respectively, at *ψ* = 0.075%. Irrespective of the nanofluid type, *h_avg_* increases with the augmentation of nanoparticle concentration and *Re*. Such enhancement in heat transfer is consistent with the literature [2,63,64,65]. However, the rate of *h_avg_* increment is highest for the single-phase and Eulerian–Eulerian models, followed by the Lagrangian–Eulerian model. The *h_avg_* of the MWCNT/GNP–H_2_O hybrid nanofluid is larger than that of the MWCNT–H_2_O nanofluid. At optimal *Re* and *ψ*, the enhancements of *h_avg_* of MWCNT/GNP–H_2_O nanofluid compared with MWCNT–H_2_O nanofluid are 14%, 12.2%, 12.9%, 13.1% and 9.1% when estimated using single, mixture, VOF, Eulerian and Lagrangian–Eulerian models, respectively.

**Figure 10:**
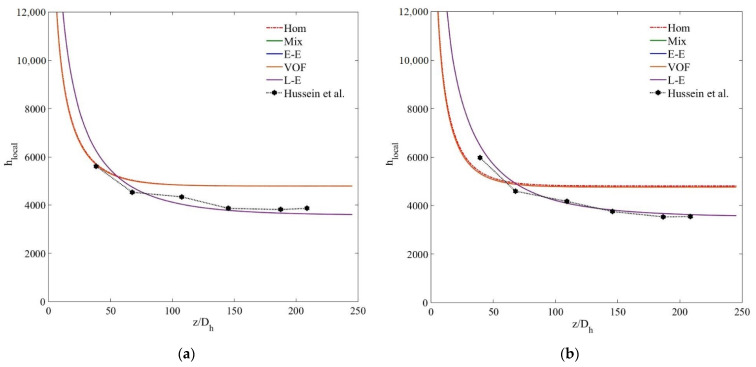
(**a**) Local heat transfer coefficient (*h_local_*) of MWCNT–H_2_O (*ψ* = 0.25%) nanofluid; (**b**) local heat transfer coefficient (*h_local_*) of MWCNT/GNP–H_2_O (*ψ* = 0.25%) nanofluid.

To further elaborate the accuracy trends of the tested models in computing the thermal characteristics of the nanofluid, Figure 10 demonstrates the local heat transfer coefficient (*h_local_*) distribution along the nondimensional streamwise wall length of the channel for MWCNT–H_2_O and MWCNT/GNP-H_2_O nanofluids at *ψ* = 0.25%. The reduction of *h_local_* from the highest value at the entrance region of the channel to subsequently stabilised low values refers to thermally and hydrodynamically developing and fully developed flow regions. In agreement with previously stated results, a comparison of the experimental and numerical data signifies the superiority of the Lagrangian–Eulerian approach in predicting the *h_local_* distribution of nanofluids over single and other multiphase models. The latter models predict significantly low *h_local_* values along *z*/*D_h_* > 75 while overestimating the results along the remaining tube length 75< *z/D_h_* < 250. For the MWCNT-H_2_O (*ψ* = 0.25%) nanofluid, the deviations of *h_local_* results determined using the single-phase, mixture, VOF, Eulerian and Lagrangian–Eulerian models are 13.3%, 11.9%, 12.6%, 11.68% and 6.85%, respectively, while for the MWCNT/GNP-H_2_O (*ψ* = 0.25%) nanofluid, these deviations become 17.5%, 14%, 14.4%, 13.92% and 5.47%, respectively. 

**Figure 11:**
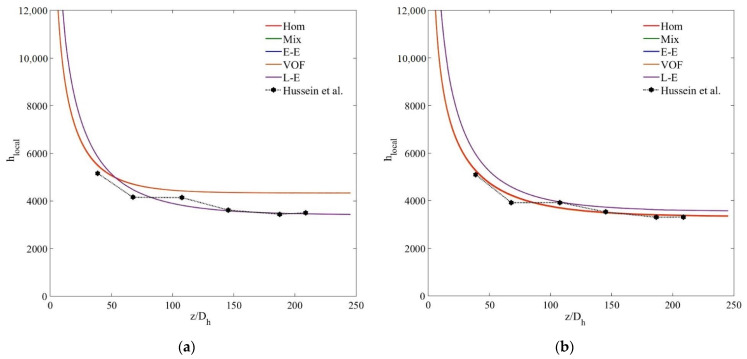
(**a**) Local heat transfer coefficient (*h_local_*) of MWCNT -H_2_O (*ψ* = 0.125%) nanofluid; (**b**) local heat transfer coefficient (*h_local_*) of MWCNT–H_2_O (*ψ* = 0.075%) nanofluid.

The *h_local_* for the small nanoparticle concentrations of 0.125 wt% and 0.075 wt% (Figure 11) also illustrate the accuracy trend of the tested models in the order of single, Eulerian–Eulerian and Lagrangian–Eulerian. However, for the highly diluted nanofluids, the tested models show quite insignificant deviations, that is, at *ψ* = 0.075%, the percentage differences between the experimental and computed *h_local_* data of single-phase, mixture, VOF, Eulerian and Lagrangian–Eulerian models are 3%, 3.2%, 4.8%, 3% and 4.7%, respectively. Thus, it can be concluded that all the single- and multiphase numerical models estimate quite accurate thermal fields for highly diluted nanofluids when the interphase interactions also become insignificant. However, single-phase and Eulerian–Eulerian models fail to predict the thermal characteristics of dense nanofluids due to the overestimation of the effective thermal conductivity and interphase interactions.

**Figure 12:**
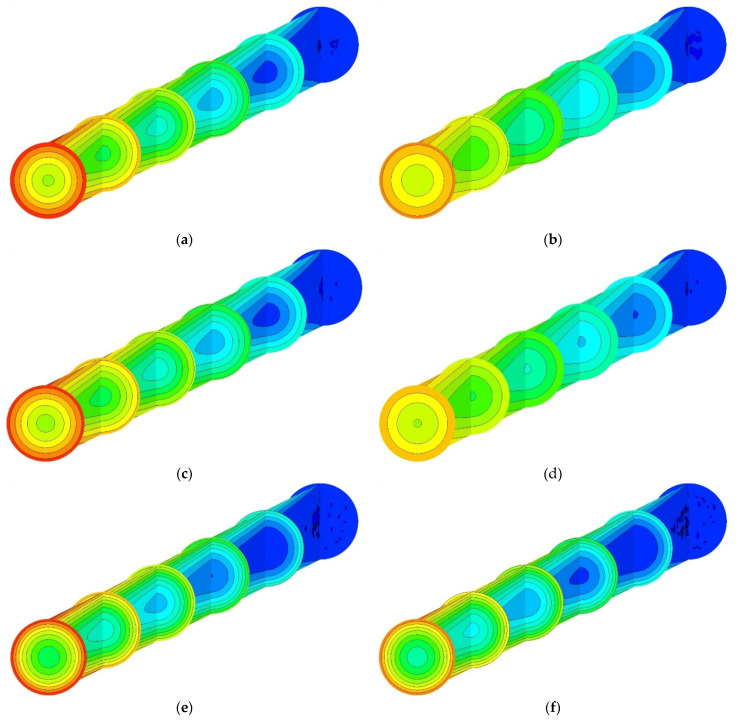
Temperature contours along six cross-sectional areas and symmetrical plane of the channel at *Re* = 470, *ψ* = 0.25%: (**a**,**b**) single-phase model, (**c**,**d**) mixture and (**e**,**f**) Lagrangian–Eulerian model.

Figure 12 depicts the temperature variation along the channel central longitudinal and cross-sectional planes (*z/D_h_* = 0, 49, 98, 147, 196, 245) for the MWCNT (left) and MWCNT/GNP (right) nanofluids at *ψ* = 0.25%. As stated earlier, the thermal contours also verify that the MWCNT/GNP–H_2_O hybrid nanofluid improves the heat transfer rate compared with the MWCNT–H_2_O nanofluid. However, this heat transfer enhancement rate predicted by different models is in the order of single-phase, Eulerian–Eulerian and Lagrangian–Eulerian models. 

### 5.3. Pressure Drop Characteristics

**Figure 13:**
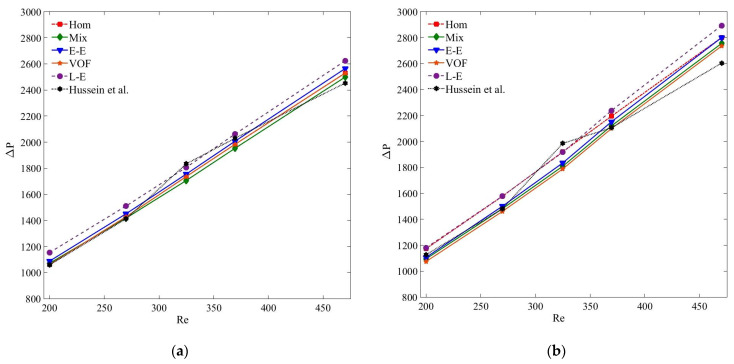
(**a**) Pressure drop (Δ*P*) of MWCNT–H_2_O (*ψ* = 0.25%) nanofluid; (**b**) pressure drop (Δ*P*) of MWCNT/GNP–H_2_O (*ψ* = 0.25%) nanofluid.

**Figure 14:**
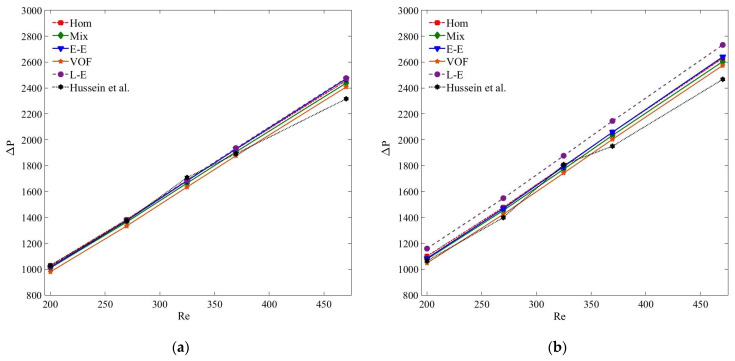
(**a**) Pressure drop (Δ*P*) of MWCNT–H_2_O (*ψ* = 0.125%) nanofluid; (**b**) pressure drop (Δ*P*) of MWCNT/GNP–H_2_O (*ψ* = 0.125%) nanofluid.

**Figure 15:**
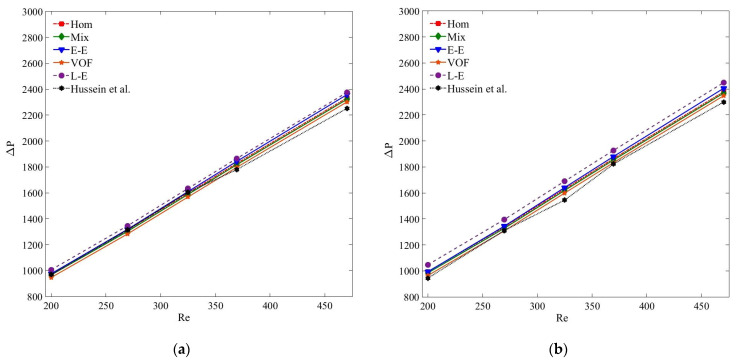
(**a**) Pressure drop (Δ*P*) of MWCNT–H_2_O (*ψ* = 0.075%) nanofluid; (**b**) pressure drop (Δ*P*) of MWCNT/GNP–H_2_O (*ψ* = 0.075%) nanofluid.

Figure 13 presents the compression of pressure drop results of MWCNT–H_2_O and MWCNT/GNP–H_2_O nanofluid at *ψ* = 0.25%. Clearly, the accuracies of the pressure drop results estimated by all the models are in acceptable range, and are in the order of single-phase, Eulerian–Eulerian and Lagrangian–Eulerian models. Of all the tested models, the Lagrangian–Eulerian approximation demonstrates the most deviated pressure drop values due to the inclusion of interphase interactions, yet the computed results are in the acceptable limit. Similarly, all the tested models determine quite similar and accurate results for the smaller nanoparticle concentration and at low *Re* (Figure 14 and Figure 15). As the nanoparticle concentration or the flow rate increases, the deviation of the computed results also increases. At *Re* = 470, the percentage errors of the single-phase, mixture, VOF, Eulerian and Lagrangian–Eulerian models are 7%, 5.7%, 6.7%, 5% and 11.1% for the MWCNT/GNP–H_2_O (*ψ* = 0.25%) nanofluid, while decreasing to 3.4%, 2.9%, 4.6%, 2.1% and 6.5% for the nanoparticle concentration of 0.075%, respectively. 

## 6. Conclusions

The homogenous, Eulerian–Eulerian (VOF, mixture, Eulerian) and Lagrangian–Eulerian approaches for simulating the hydrothermal characteristics of MWCNT–H_2_O and MWCNT/GNP–H_2_O hybrid nanofluids are assessed by incorporating the influence of nanolayering around the nanoparticles. The analysis is performed for the laminar forced convection of nanofluids in a minichannel subjected to constant heat flux by considering the nanoparticle weight concentration and Reynolds number of 0.075–0.25% and 200–470, respectively. The results are evaluated in terms of average and local convective heat transfer coefficients, thermal contours and pressure drop across the channel. The study findings can be concluded as follows.

- Single and all the multiphase numerical models estimate reasonably accurate convective heat transfer coefficient results for diluted nanofluids and at low Reynolds number when the interphase interactions are negligible.
- The single-phase and Eulerian–Eulerian models overestimate the thermal fields of the nanofluid with a more perceptible difference at high nanoparticle concentration and Reynolds number.
- The multiphase Eulerian–Eulerian approaches reveal marginal differences between average and local convective heat transfer coefficients results. The maximum deviation of the single-phase, VOF, mixture and Eulerian models are 10.73%, 9.5%, 9% and 9.4%, respectively, for the average heat transfer coefficient results of the MWCNT/GNP–H_2_O (0.25 wt%) nanofluid at Reynolds number of 470.
- Of all the tested models, the Lagrangian–Eulerian approximation of nanofluid provides the most accurate convective heat transfer coefficient with a maximum deviation of 5.34% for 0.25 wt% of MWCNT–water nanofluid at Reynolds number of 470. However, the model requires high memory and computational time to compute the trajectories of individual nanoparticles.
- Despite the thermal discrepancies, all numerical models determine quite accurate pressure drop results at all the studied nanoparticle concentrations and Reynolds numbers.

## Figures and Tables

**Table 1:** Thermophysical properties of multiwalled carbon nanotubes (MWCNT) and graphene nanoplatelets (GNP) nanoparticles.

Material	$\rho \left[kg\;m^{-3}\right]$	Cp $\left[J\;kg^{-1}K^{-1}\right]$	k $\left[W\;m^{-1}K^{-1}\right]$	Ref.
MWCNT	2100	630	1500	[57]
GNP	2200	790	3000	[45]

**Table 2:** Specifics of tested grid sizes.

Grid	Δt	$N_{t}$
G1	0.058	826,541
G2	0.044	1,102,676
G3	0.031	1,442,556
G4	0.023	2,302,986

## Data Availability

Data is available within the article.

## Nomenclature

AL_2_O_3_	Aluminium oxide (-)	$P_{r}$	Prandtl number (-)
$C_{c}$	Cunningham correlation (-)	Q	Heat flux (${W\;m}^{-2}$)
$C_{D}$	Drag coefficient (-)	Re	Reynolds number (-)
$C_{f}$	Inertial coefficient (-)	$S_{m}$	Momentum source term (W/m^3^)
$C_{p}$	Specific heat (${\mathrm{Jkg}}^{-1}K^{-1}$)	$S_{e}$	Energy source term (N/m^3^)
Cu	Copper (-)	T	Temperature (K)
d	Diameter (m)	TiO_2_	Titanium dioxide (-)
$d_{ij}$	Deformation tensor (s^−1^)	t	Time (s)
$D_{h}$	Channel hydraulic diameter (m)	Δt	Number of cells (-)
E-E	Eulerian model (-)	V	Velocity (${m\;s}^{-1}$)
$F_{drag}$	Drag force (ms^−2^)	VOF	Volume of fluid model
$F_{lift}$	Lift force (ms^−2^)	Y+	Wall distance of initial node (-)
$F_{col}$	Collision force (ms^−2^)	**Greek Symbols**	
$F_{gravity}$	Force due to gravity (ms^−2^)	*μ*	Viscosity (${\mathrm{kg}\;m}^{-1}\;s^{-1}$)
$F_{Brownian}$	Brownian force (ms^−2^)	β	Nanoparticle aspect ratio (-)
$F_{thermophoresis}$	Thermophoresis force (ms^−2^)	₸	Stress-strain tensor (-)
$F_{virtual}$	Virtual mass force (ms^−2^)	*ρ*	Density (${\mathrm{kg}\;m}^{-3}$)
$F_{pressure}$	Pressure force (ms^−2^)	ψ	Particle weight fraction (-)
g	Gravitation acceleration (${\mathrm{ms}}^{-2}$)	*φ*	Particle effective volume fraction (-)
GNP	Graphene nanoplatelets	$\eta_{r}$	Relative viscosity of nanofluid (-)
H	Height (m)	ϖ	Particle sphericity
*h*	Convective heat transfer coefficient (W m^−2^K^−1^)	v	Kinematic viscosity (${\mathrm{ms}}^{-1}$)
Hom	Single-phase model (-)	**Subscripts**	
*M*	Mass (kg)	avg	Average
*Mix*	Mixture model (-)	f	Liquid phase
$K_{n}$	Knudsen number (-)	h	Hydraulic diameter
*k*	Thermal conductivity (${\mathrm{Wm}}^{-1}K^{-1}$)	in	Inlet
$K_{B}$	Boltzmann constant (${\mathrm{JK}}^{-1}$)	local	Local
L	Channel length (m)	m	Mixture
L-E	Lagrangian–Eulerian model (-)	n	Nanoparticles
N	Avogadro’s number (-)	nf	Nanofluid
Nu	Nusselt number (-)	r	Relative
M	Molar mass (${\mathrm{kg}\;\mathrm{mol}}^{-1}$)	s	Nanoparticle phase
MWCNT	Multiwalled carbon nanotubes (-)	T	Reference temperature
P	Pressure (${\mathrm{Nm}}^{-2}$)	t	Total
PEC	Performance evaluation criteria (-)	out	Outlet
n	Particle shape factor	w	Wall
